# Biotinidase Activity Inhibition as a Biomarker of Effect to Mercury: Evidence from Amazonian Riverside Populations, In Vitro Assays, and In Silico Analyses

**DOI:** 10.1021/acsomega.5c12680

**Published:** 2026-06-08

**Authors:** José Luiz Martins do Nascimento, Gabriela de Paula Arrifano, Robson Nascimento Viana, Marcus Augusto-Oliveira, Amanda Lopes-Araújo, Leticia Santos-Sacramento, Victor Cezar Gomes Melo, Rafaela Silva de Sousa, Carlos Gabriel da Silva de Souza, Chubert Bernardo Castro de Sena, Luiz Carlos Santana da Silva, Maria da Conceição Nascimento Pinheiro, Jeronimo Lameira, Maria Elena Crespo-López, Barbarella Matos Macchi

**Affiliations:** † Laboratório de Neuroquímica Molecular e Celular, 37871 ICB-UFPA, Rua Augusto Corrêa 1, 66075-110 Belém, PA, Brazil; ‡ Programa de Pós-Graduação em Farmacologia e Bioquímica, 37871 ICB-UFPA, Rua Augusto Corrêa 1, 66075-110 Belém, PA, Brazil; § Laboratório de Farmacologia Molecular, 37871 ICB-UFPA, Rua Augusto Corrêa 1, 66075-110 Belém, PA, Brazil; ∥ Laboratório de Erros Inatos do Metabolismo, 37871 ICB-UFPA, Rua Augusto Corrêa 1, 66075-110 Belém, PA, Brazil; ⊥ Laboratório de Modelagem Molecular, ITEC-UFPA, Rua Augusto Corrêa 1, 66075-110 Belém, PA, Brazil; # Laboratório de Biologia Estrutural, 37871 ICB-UFPA, Rua Augusto Corrêa 1, 66075-110 Belém, PA, Brazil; ∇ Núcleo de Medicina Tropical, UFPA, Av. Generalíssimo Deodoro 92, 66055-240 Belém, PA, Brazil; ○ Instituto Nacional de Ciência e Tecnologia em Neuroimunomodulação (INCT-NIM), Av. Brasil, 4365, 21040-900 Rio de Janeiro, RJ, Brazil

## Abstract

Mercury is one of the most toxic substances found in nature and can be harmful to both the environment and human health. The brain is a critical organ that is sensitive to mercury exposure, especially during development, when it can cause neurological dysfunction and abnormalities as well as other neurodevelopmental deficits. In this context, the identification of new biomarkers of mercury exposure and intoxication will provide valuable tools for the prevention, response, and treatment of environmental contamination. In this study, we propose biotinidase activity as a biomarker of effect, which may provide a direct measure of mercury intoxication, by performing a complete screening in silico, in vitro, and in vivo in human blood samples. Biotinidase is an enzyme that is responsible for the recycling of biocytin. A deficiency of biotinidase is an autosomal recessive disorder that has a variable clinical expression, including delays in development. We evaluated biotinidase activity in the blood of Amazonian riverine populations environmentally exposed to mercury compared to those not exposed. Also, in vitro, other metals were tested to verify the mercury specificity and selectivity for the inhibition of the biotinidase activity. An in silico molecular docking analysis of the target enzyme was performed to understand the preference of methylmercury for the catalytic site. We found that mercury exposure decreases serum biotinidase activity, while other metals do not change it. The molecular docking analysis of the active site indicated a high level of interaction with nucleophiles, with the thiol and selenic groups forming strong bonds with mercury, which implies that cysteine is a potential target for the inhibitory action of mercury. Overall, the evidence indicates that biotinidase activity may serve as a novel biomarker of effect for the assessment of mercury intoxication, which constitutes a persistent threat to both the environment and human populations in the Amazon region.

## Graphical abstract

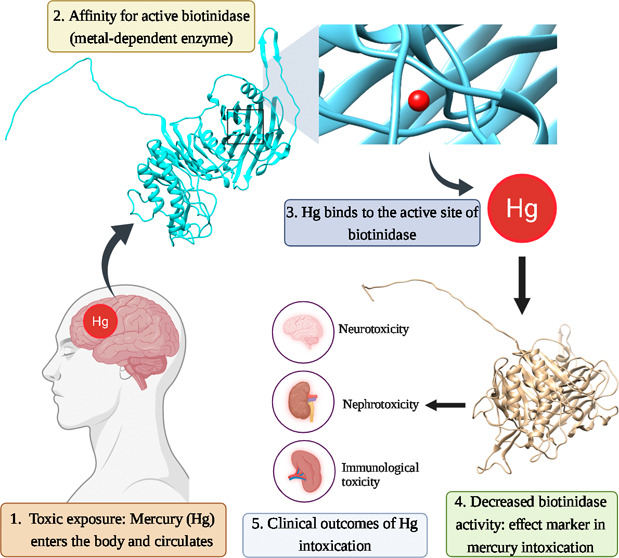

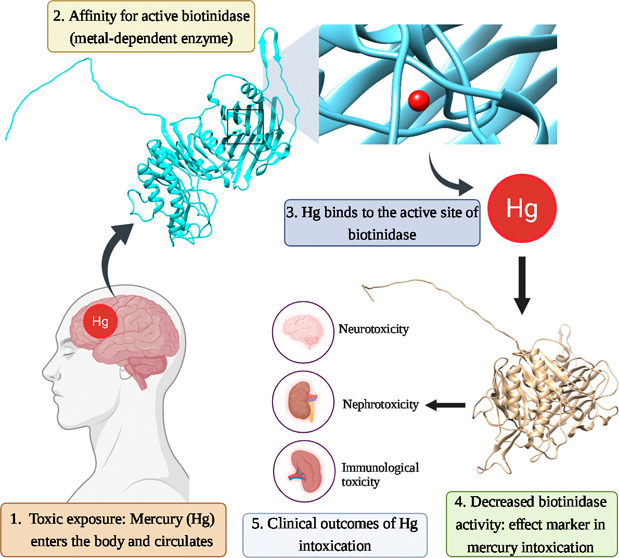

## 1 Introduction

Mercury is a highly stable and nondegradable metal, and exposure to this potentially toxic element is a major global public health issue.^1–4^ Globally, emissions of mercury resulting from anthropogenic activities have increased substantially in recent decades, contributing to widespread environmental distress. In the Amazon region, mercury levels in the environment often exceed the secure limits recommended by health agencies, resulting in high levels of bioaccumulation and the contamination of fish, which, in turn, affects local riverside communities.^5^

A number of epidemiological studies have explored the relationship between exposure to mercury and the health of the residents of these communities. Even low levels of exposure to mercury raise concerns due to the genotoxic and mutagenic effects of this metal, especially as many Amazonian populations undergo chronic exposure to this toxin.^6–11^ Mercury may cause a range of diseases including neurological and skin disturbances, leading to symptoms such as tremors, memory loss, ataxia, skin rashes and swelling, dermatitis, and acrodynia, among others.^12^ Particularly during periods of rapid brain development, mercury leads to profound neurological problems, which are manifested in delays in the acquisition of motor skills and language, as well as genotoxicity and other neurodevelopmental deficits.^13–18^

Given this, the identification of reliable biomarkers that can detect the effects of mercury exposure will be crucial for the understanding of the potential health risks, monitoring disease progression, and evaluating possible treatment options in the region.^10,19,20^ Here, we propose the enzyme biotinidase (BTD) as a candidate biomarker of mercury exposure because the deficiency of such an enzyme causes neurological and skin disorders similar to mercury ones.

BTD (EC 3.5.1.12) catalyzes the conversion of biocytin to free biotin by removing a lysine group. Biotin is a critical cofactor for a number of different carboxylase enzymes, including holocarboxylase synthetase, which binds biotin to apocarboxylases to form active enzymes.^21–23^

The BTD enzyme consists of 543 amino acids and contains six potential N-glycosylation sites. Computational models indicate that the structure comprises two separate domains. Domain A, which is homologous to the nitrilases/amidases, is where the catalytic triad (glutamate 112, lysine 212, and cysteine 245) is located, while domain B is similar to the anchoring domain of Vanin-1 and the Fhit domain of NitFhit and may play a role in biotin binding. These two domains are connected by a flexible region.^24,25^ The present study evaluated the inhibition of BTD activity as a potential biomarker of effect to mercury exposure by integrating evidence from Amazonian riverside populations, controlled in vitro biochemical assays, and in silico molecular interaction analyses.

## 2 Results

### 2.1 Biotinidase Activity in Amazonian Riverine Populations

In the present study, a total of 318 individuals were sampled from the four riverside communities surveyed in eastern Pará. The demographic characteristics and biochemical profiles of the participants are summarized in Table 1.

**Table 1:** Demographic Characteristics of the Four Amazonian Riverside Populations Surveyed in the Present Study and Their Biochemical Profiles

	PNC	SLT	BRR	TUC
number of subjects	16	71	72	155
gender female, %	100	73.2	81.3	59.6
age, y	37 (30–43)	40 (29–52)	48 (39–61)^a^	42 (32–56)
biotinidase activity, nmol·ml^–1^·min^–1^	6.74 (5.09–9.19)	4.46 (2.08–7.33)^b^	4.48 (1.39–9.59)^b^	3.31 (2.51–3.91)^b^

a *P* < 0.05 vs all other groups in the same line; Dunn’s post hoc test for the Kruskal–Wallis nonparametric analysis of variance.

b Below reference values of 5.0–10.0 nmol·ml^–1^·min^–1^ reported in the literature.

The parameters are presented as the median and interquartile range, except for biotinidase activity, which is presented as the mean and minimum–maximum values. A majority of the individuals in the São Luiz do Tapajós (SLT), Barreiras (BRR), and Tucuruí Lake (TUC) populations had reduced biotinidase activity (<5 nmol·ml^–1^·min^–1^), while all the individuals from Panacauera (PNC) had normal levels of biotinidase activity (≥5 nmol·ml^–1^·min^–1^) (Figure 1). No significant difference was found in biotinidase activity between the two genders (data not shown).

**Figure 1:**
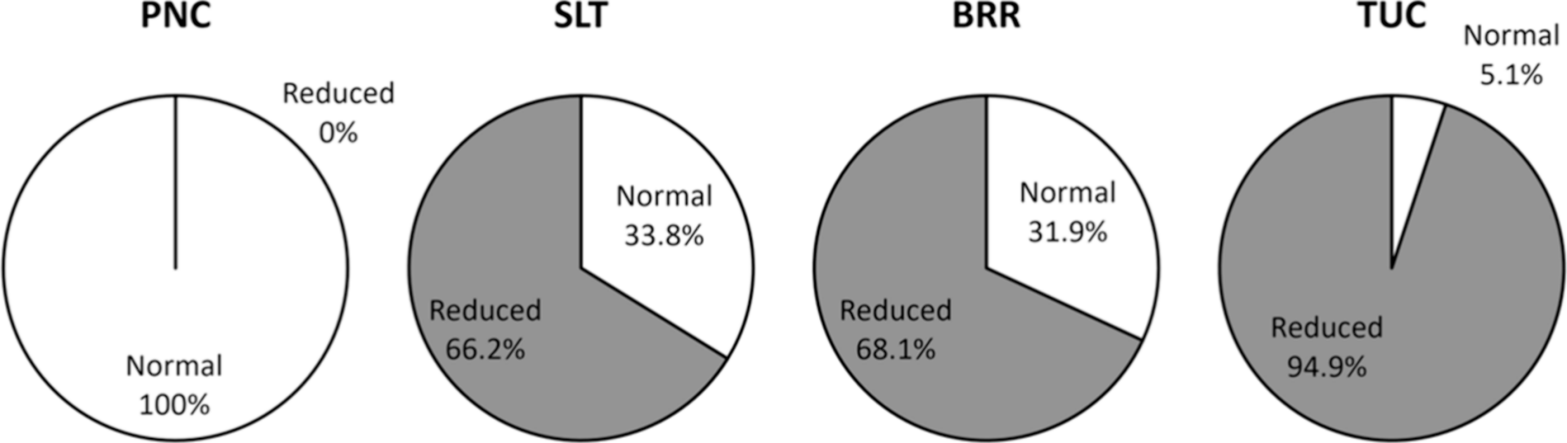
Prevalence (%) of subjects presenting reduced activity of the BTD enzyme in each of the four study communities analyzed in the Brazilian state of Pará. Normal biotinidase enzyme (≥5 nmol·ml^–1^·min^–1^) and reduced biotinidase enzyme (<5 nmol·ml^–1^·min^–1^).

Biotinidase is a protein synthesized by the liver and secreted into plasma. Considering this, possible liver dysfunction was ruled out in communities that presented reduced biotinidase activity due to normal levels of gamma-glutamyl transferase (GGT), aspartate aminotransferase (AST), and alanine aminotransferase (ALT) (Table 2).

**Table 2:** Biochemical Indicators of Liver Function in Populations Exhibiting Low BTD Activity^a^

	SLT	BRR	TUC
GGT, U/l	17.9 (11.5–24.3)	18.9 (13.3–25.5)	32 (24–42)
AST, U/l (TGO)	17.5 (14.9–19.2)	15.7 (14.0–18.4)	23 (19–27)
ALT, U/l (TGP)	10.0 (7.5–13.5)	11.6 (7.7–15.5)	9 (5–16)

a Reference values: ALT, 7–56 U/L; AST, 10–40 U/L; GGT, 5–40U/L.

The mean BTD activity in the individuals from PNC, an area described in the literature as nonexposed to mercury, was significantly higher (*P* < 0.001) than the values recorded in the other three populations, which have been reported as mercury-exposed based on hair total mercury concentrations by Pinheiro et al. (2006 and 2008) and Arrifano et al. (2018). Among the mercury-exposed locations, the BTD activity recorded at TUC was significantly lower (*P* < 0.001) than both SLT and BRR (Figure 2).

**Figure 2:**
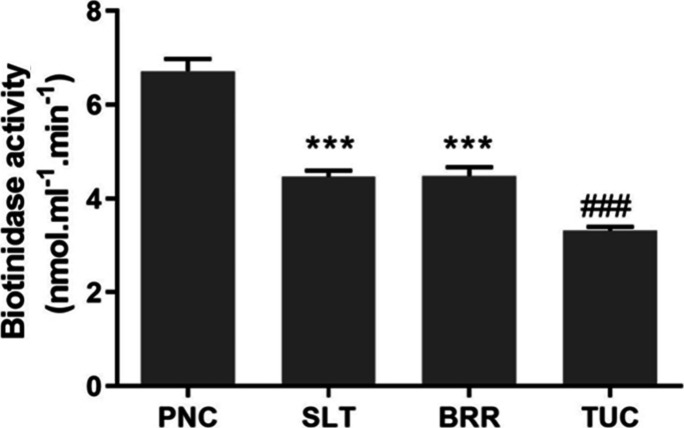
Biotinidase activity recorded in the present study in the populations of PNC, SLT, BRR, and TUC. ***P < 0.001 for Tukey’s post hoc test, in comparison with PNC; ###*P* < 0.001 for Tukey’s post hoc test, in comparison with all other communities.

### 2.2 Biotinidase Activity and Human Mercury Exposure

In order to investigate the relation between biotinidase activity and mercury exposure, we also collected hair samples from 132 individuals from the Tucuruí Lake community that have agreed to donate hair for measuring mercury concentration. Demographic characteristics and biochemical and mercury exposure profiles of the Tucuruí population are described in Table 3.

**Table 3:** Demographic Characteristics and Biochemical and Mercury Exposure Profiles of the Tucuruí Population^a^

**TUC^b^** (*n* = 132)	
**Characteristics**	
female, *n* (%)	87 (59.18%)
age, years	43 (32–56)
weight, kg	62.3 (55.2–72.7)
height, cm	154.0 (150.0–161.0)
BMI, kg/m^2^	25.9 (23.1–29.2)
**Biochemical profile**	
biotinidase activity, nmol·ml^–1^·min^–1^	3.21 (2.55–3.93)
**Hair mercury concentration**	
mercury, μg/g	9.28 (4.74–15.12)

a The parameters are presented as the median and interquartile range, except for biotinidase activity, which is presented as the mean and minimum-maximum values.

b The TUC community presented a median hair mercury concentration of 9.28 μg/g (interquartile range: 4.74–15.12), indicative of substantial chronic exposure, along with a mean BTD activity of 3.21 nmol·mL^–1^·min^–1^, below the reference ranges reported for unexposed groups.

### 2.3 Hair Mercury Levels and Their Relationship with Biotinidase Activity

Mercury hair concentration ranged up to a maximum value of 27.42 μg/g (Table 4). To analyze the possible relationship between mercury exposure and biotinidase activity, participants were distributed according to hair mercury levels (<10 μg/g or ≥10 μg/g) (Table 4). Although we found no difference between the two groups, there was only a discrete reduction in the mean BDT activity in the subgroup with lower levels of Hg contamination, and no significant shifts in any of the variables were examined. This lack of a systematic pattern may be due to the fact that the mercury content of the hair may not be representative of the actual exposure of the individual.

**Table 4:** Profile of Participants According to Hair Mercury Levels (<10 μg/g or ≥10 μg/g)^a^

	**<10 μg/g of THg** *n* = 67	**≥10 μg/g of THg** *n* = 65	P*
**Characteristics**			
female	39 (58.20%)	35 (53.84%)	
age, years	42 (28–53)	42 (32–55)	0.4368
weight, kg	60.6(55.0–70.6)	63.9(55.6–72.6)	0.5822
height, cm	153.0 (150.0–160.0)	154.0 (150.0–163.5)	0.1910
BMI, kg/m^2^	26.2 (22.8–29.2)	25.2 (23.2–28.9)	0.5916
**Biochemical profile**			
biotinidase activity, nmol·ml^–1^·min^–1^	3.37 (2.62–3.99)	3.16 (2.39–3.99)	0.4661
**Hair mercury concentration**			
mercury, μg/g	5.16 (3.31–7.34)	15.12 (12.24–18.21)	<0.0001

a The parameters are presented as the median and (interquartile range), except for Biotinidase activity, which is presented as the mean and (minimum–maximum) values. *P < 0.05 vs other group in the same line; P* Mann–Whitney test.

### 2.4 In Vitro Inhibition of Biotinidase by Mercury

To account for the differences observed among the study populations and the potential inhibitive influence of mercury on BTD activity, in vitro assays were conducted on blood samples of healthy donors with no known exposure to mercury (Figure 3). The analysis of the varying concentrations of four different metals found a significant dose-dependent response only between BTD activity and mercury. While a significant reduction in BTD activity was observed at high MeHg concentrations (Figure 3, top left panel), no clear pattern was found (Figure 3top right, bottom left, and bottom right panels) in any of the other metals tested (ZnCl_2_, CuCl_2_, and FeCl_2_). No significant change in enzyme activity was detected at MeHg concentrations of 0.005 and 0.05 μM, although enzyme activity declined progressively from 0.5 μM MeHg onward. Biotinidase activity decreased by 20% at a MeHg concentration of 0.5 μM, 55% at 5 μM, and 82% at 50 μM, reflecting the significant impact of mercury on the catalytic function of the enzyme.

**Figure 3:**
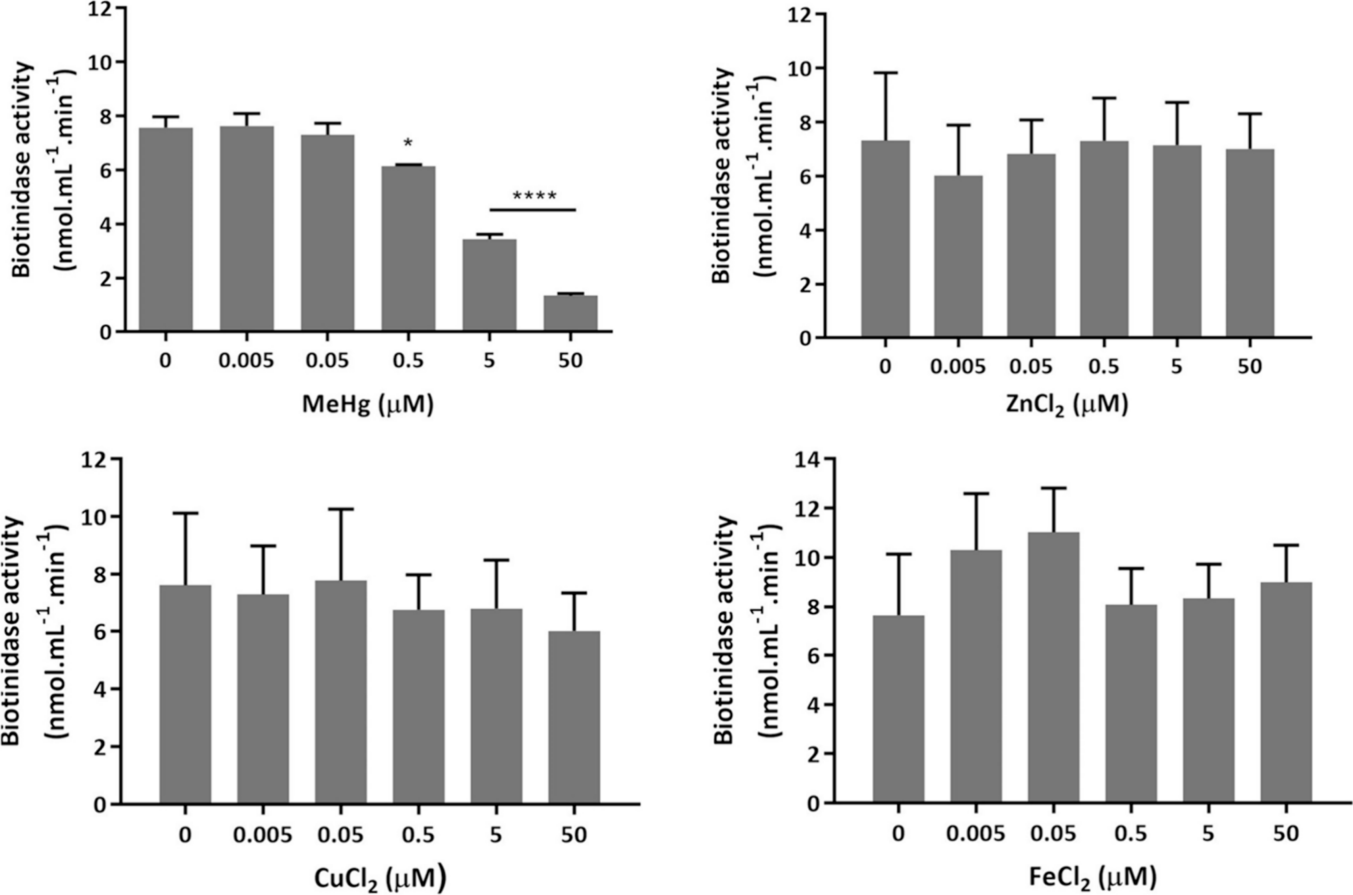
Serum biotinidase activity following incubation with different metallic compounds at varying concentrations: (top left) Methylmercury chloride (MeHg); (top right) Zinc chloride (ZnCl_2_); (bottom left) Copper­(II) chloride (CuCl_2_); (bottom right) Iron­(II) chloride (FeCl_2_). In the specific case of MeHg, significant differences in BTD activity were found between concentrations: *P < 0.05 difference versus 0.005 μM and 0.05 μM, and ****P < 0.001 difference versus all other groups.

### 2.5 Structural Modeling of Biotinidase

To further elucidate how mercury exposure reduces biotinidase activity, we first obtained the three-dimensional structure of biotinidase by using homology modeling. The best model of biotinidase (Figure 4A) made with Swiss-Model had 91.99% of its residues contemplated in the Ramachandran plot (Figure 4B) and a well-conserved active site. This model was used for docking analysis.

**Figure 4:**
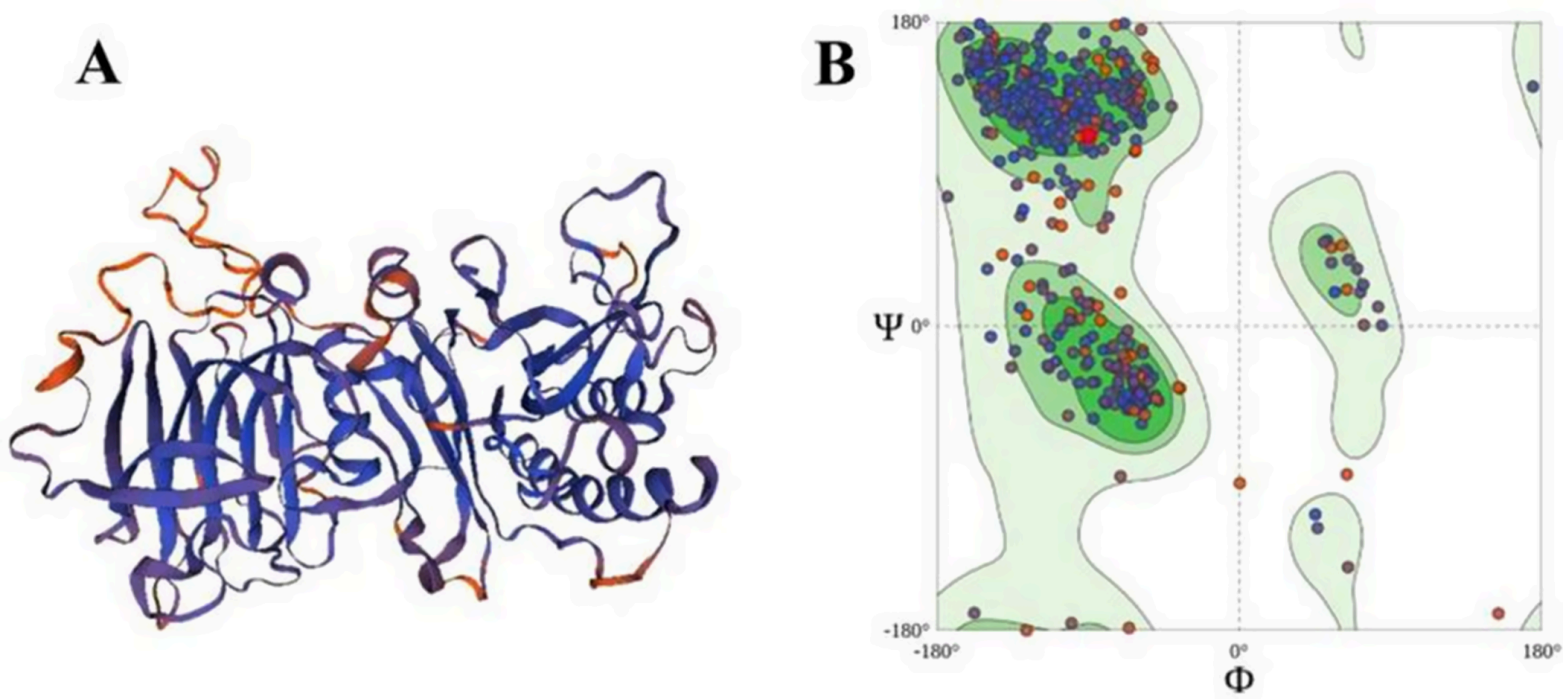
(A) 3D structure of the biotinidase 4CYF model produced in Swiss-Model; (B) Ramachandran plot of the model.

### 2.6 In Silico Interaction between Biotinidase and Methylmercury

The docking analysis returned seven results (Table 5). The meticulous relative analysis of the MolDock and Rerank scores identified pose 06 as the best docking pose. The results of this analysis indicate that MeHg interacts with Cys245 at a distance of 3.301 Å (Figure 5). The observed reduction in BTD activity with increasing MeHg concentrations (Figure 3A) is consistent with the findings of this in silico analysis, which modeled the interaction between MeHg and the catalytic site of the biotinidase.

**Table 5:** Docking Results

pose ID	**MolDock score**	**rerank score**	**distance (Å)**	**cysteine contribution**
[00]	–18.0724	–13.8641	3.313	–0.4949
[02]	–17.4401	–12.3302	3.319	–0.8719
[01]	–17.4321	–12.7597	3.512	–0.8043
[03]	–15.7151	–12.0261	3.304	–0.6035
[04]	–15.3849	–11.6555	3.301	–0.6851
[06]	–15.3466	–12.0662	3.301	–1.0225
[05]	–15.3317	–11.5469	4.697	–1.0199

**Figure 5:**
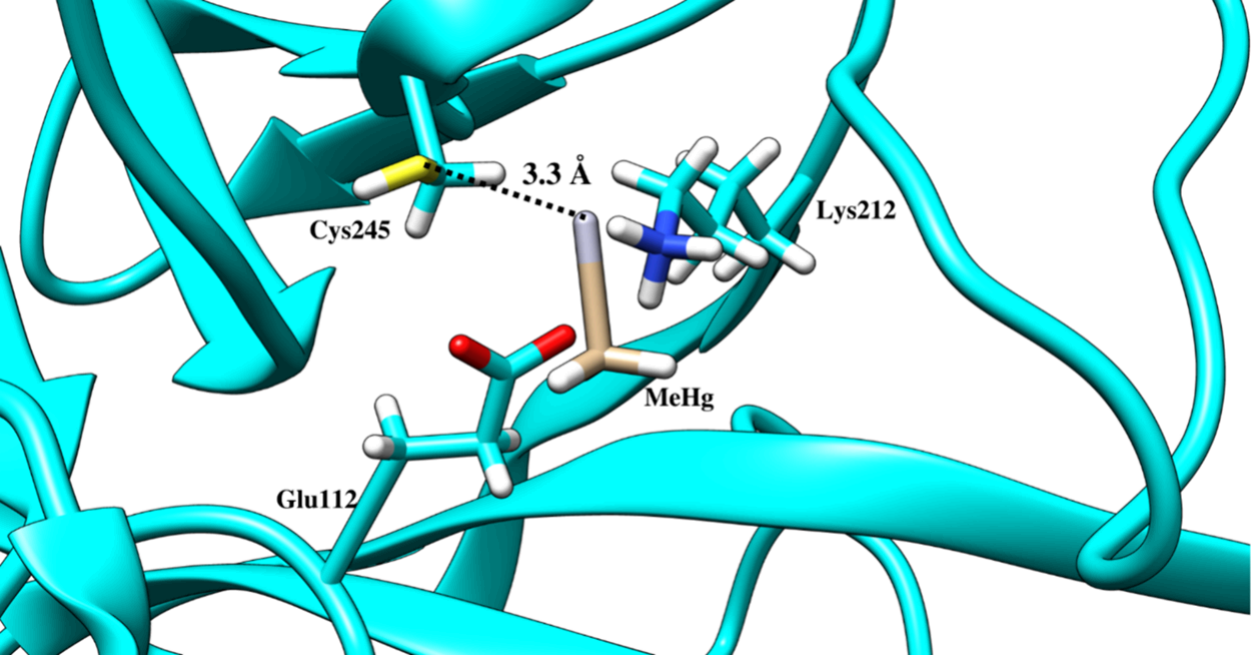
Relative distance of MeHg-Cys245 S, showing cysteine in yellow and methylmercury shown in brown and silver; lysine in dark blue and glutamic acid in red. Produced in UCSF Chimera.^26^

## 3 Discussion

The present work shows for the first time a potential role for the biotinidase (BTD) enzyme as a biomarker of effect in mercury-exposed Amazonian riverside populations. BTD contains a sulfhydryl group and is responsible for the recycling of endogenous biotin, a substance that is vital for the synthesis of a number of different proteins, including histones and multiple carboxylases that are involved in key metabolic processes, such as fatty acid synthesis, the catabolism of a number of branched-chain amino acids, and energy production.^27–32^ Given its structure, biotinidase can be sensitive to chemical modifications caused by environmental contaminants.^24,33^ In particular, heavy metals, such as mercury, have been shown to inhibit metabolic enzymes, having a significant impact on a number of different metabolic processes, which provokes neurological impairment.^34–38^

### 3.1 BTD Activity in Amazonian Riverine Populations

Populations in the Amazon region, in particular those of the riverside communities that depend on fish for their subsistence, have been exposed chronically to mercury in the environment for decades, raising significant concerns for both the environment and public health, primarily through the consumption of contaminated fish and activities such as gold mining.^6,7,9,10,39–44^ The high levels of mercury found in these populations further reinforce the need for the identification of reliable biomarkers of the impacts of contamination (i.e., biomarker of effect). The early detection of problems using these biomarkers will be crucial for the adequate assessment of health risks and the implementation of effective preventive measures to preempt adverse effects before they become irreversible.

In this sense, BTD activity is one potential biomarker for the assessment of the effects of exposure of humans to mercury, given the involvement of this enzyme in essential metabolic pathways and its susceptibility to toxicological disruptions. The assessment of this enzyme may provide valuable insights into the impact of mercury as a pollutant given its potential to induce significant biological alterations.

The investigation of four Amazonian populations revealed that BTD activity was reduced significantly in the three communities—São Luiz do Tapajós, Barreiras, and Tucuruí Lake—most exposed to mercury, while the Panacauera population, which is considered not exposed,^6,7,9,10^ presented BTD activity within the normal range, based on the published reference values of 5.0–10.0 nmol·mL^–1^·min^–1^.^32,45,46^

The observed reduction in BTD activity in exposed populations may result from multiple mechanisms including decreased enzyme synthesis or direct inhibition of its activity by mercury. In the present study, we did not evaluate biotinidase expression levels; therefore, it is not possible to determine whether the reduced activity reflects impaired enzyme production or a direct interaction with mercury. However, our in vitro findings, demonstrating a dose-dependent inhibition of BTD activity by methylmercury, support the hypothesis of a direct inhibitory effect on the enzyme. It is also plausible that indirect mechanisms such as alterations in gene expression or protein synthesis may contribute to this effect. Regardless of the underlying mechanism, the consistent reduction in BTD activity observed across the exposed groups reinforces its relevance as a functional biomarker of mercury exposure.

Mercury toxicity can have a significant impact on biotinidase activity in the body, primarily through its interaction with the essential sulfhydryl groups of the enzyme.^47–50^ Mercury, in both its organic and inorganic forms, can inhibit biotinidase activity by binding to these sulfhydryl groups, thereby disrupting enzyme function.^27^ This inhibition can lead to a cascade of metabolic disturbances, given that a deficiency of biotinidase impairs the ability of the body to recycle biotin, which affects the activity of the carboxylase enzyme with potential neurological and systemic consequences.^30,46,51,52^

Given this, the reduced BTD activity detected in the populations exposed to mercury reinforces the conclusion that this metal may have a direct effect on the enzyme, irrespective of liver dysfunction, according to the findings of previous epidemiological studies, which have demonstrated protein alterations in populations exposed chronically to mercury.^6,7,53–55^ This inhibition could potentially be both a new biochemical mechanism for mercury neurotoxicity and provide an indirect indicator of mercury intoxication in impacted populations.

Interestingly, the analysis of the total mercury levels in the hair revealed a median concentration of 9.28 μg/g, reaching values as high as 27.42 μg/g in some individuals, which exceeds the thresholds established by health agencies, showing that the studied populations are among the higher mercury-exposed populations around the world.^40^ Although we could not find a significant correlation between hair mercury level and BTD activity, the TUC setting showed a lower BTD activity as compared to the other settings BRR and SLT (both located in the Tapajós area), previous works have shown that the mean mercury concentration between riverine people from Tapajós is significantly lower than Tucuruí.^56^ Also, the accumulation of mercury in the hair is widely used as a biomarker of long-term exposure; it may not provide an accurate measure of systemic mercury levels or the direct biochemical effects of this metal on enzymes.^19,20,57,58^

As hair incorporates mercury primarily during the synthesis of keratin, its concentration may not represent the variation in the distribution, metabolism, and excretion of the metal that influences its bioavailability and interaction with the enzymes circulating in the body. This may account for the fact that while biotinidase activity tended to be reduced in individuals with higher mercury levels, no significant correlation was found. Nevertheless, the observed pattern indicates that chronic exposure to mercury may contribute to the inhibition of biotinidase, which reinforces the need for further studies with larger sample sizes and additional biomarkers and blood mercury measurements to provide a better understanding of this relationship.^59,60^

This work has some limitations, for instance, (i) the limited sample size in the PNC setting; however, as discussed above, this group served only to show that Amazonian populations may show reference levels of the BTD activity; (ii) the PNC group consists of only women; although the literature does not point to a gender effect in BTD activity, sex was evaluated in this work and did not show a significant association with the enzyme in our data; (iii) the inability to establish the temporal course of mercury-induced changes in BTD activity or assess its reversibility, particularly in relation to its proposed use as a marker of mercury exposure effects; (iv) absence of blood mercury measurements, which should be included in future studies to strengthen exposure assessment. Taken together, these limitations point to the need for further study to better describe the dynamics of BTD activity in humans exposed chronically to mercury.

### 3.2 In Vitro BTD Activity

The results of the in vitro assays demonstrated a clear dose-dependent inhibition of biotinidase activity by methylmercury (MeHg). While enzyme activity remained unchanged at lower concentrations (0.005 and 0.05 μM), a significant and progressive reduction was observed at higher concentrations, ranging from 20% at 0.5 μM to 82% at 50 μM. By contrast, none of the other metals tested (Fe^2+^, Zn^2+^, and Cu^2+^) had any significant effect on biotinidase activity, which further reinforces the existence of a specific interaction between mercury and the enzyme. Hayakawa and Oizumi^27^ obtained similar findings, with mercuric agents inhibiting BTD activity at concentrations of as low as 0.005 mM.

Direct blood concentrations of MeHg exceeding 0.5 μM (∼40 μg/L) indicate advanced toxicity, manifesting clinical symptoms such as tremors and ataxia, reflecting a significant systemic burden. In contrast, reduced BTD activity detects subclinical effects at lower, environmentally relevant exposures typical of fish-consuming communities.

Total mercury levels in hair are a reliable indicator of long-term mercury exposure. In contrast, MeHg, the predominant and most toxic form of mercury, has been used in in vitro assays to investigate its direct inhibitory effects on BTD activity, as it is the biologically relevant species in human exposure in these communities.

### 3.3 In Silico BTD and MeHg Interaction

As MeHg is an electrophile, it interacts with the nucleophiles in biological systems. In particular, the thiol and selenic groups form strong bonds with mercury, which implies that cysteine is a potential target for inhibition.^13,61,62^ The presence of essential sulfhydryl residues at the active center of biotinidase makes it susceptible to mercury-induced inhibition, which can lead to reduced enzyme activity and subsequent metabolic disruptions.^25,27^ In this context, in silico molecular docking studies can provide a detailed understanding of the interactions between mercury (and other heavy metals) and biotinidase at the molecular level.

As the precise targets of MeHg are not yet fully understood,^63^ it is hoped that the present study will contribute to the understanding of the role of MeHg in any specific Cys245 inhibition mechanism. While molecular docking provides a promising starting point, these efforts must be complemented with experimental data.^64^

The results of the present study indicate that MeHg has a strong affinity with the catalytic site of the biotinidase enzyme, interacting in particular with the cysteine residues found within the active domain. This supports the conclusion that MeHg binds to the thiol (−SH) groups, which disrupts the enzyme’s normal function and leads to reduced catalytic activity.

In fact, a number of studies have shown that mercury binds preferentially to thiol (−SH) groups, leading to the inhibition of the enzyme and structural alterations.^47–50^ Similar inhibitory effects have been observed in other thiol-dependent enzymes, such as catalase, superoxide dismutase, glutathione peroxidase, and thioredoxin reductase, which play key roles in antioxidant defense.^7,36,65,66^ Other heavy metals, such as cadmium and lead, are also known to interact with the sites of critical enzymes, leading to functional impairment and metabolic imbalances.^57,66^

Together with its neurotoxic effects, the ability of mercury to accumulate in tissues exacerbates these symptoms, contributing to neurological damage and systemic health issues.^67,68^ The inhibition of biotinidase by exposure to mercury may have serious implications for the health of an individual, particularly in terms of metabolic and neurological deficits.^23,30,52,69^ Biotinidase plays a crucial role in the recycling of biotin, which is a cofactor for the carboxylases involved in key metabolic pathways, including fatty acid synthesis and gluconeogenesis.^31^

The inability of the brain to recycle biotin independently makes it particularly vulnerable to a biotinidase deficiency. Mercury-induced inhibition of biotinidase may reduce pyruvate carboxylase activity in the brain and lead to an accumulation of lactate, which would account for the neurological symptoms observed in the exposed individuals,^17,70,71^ including those of the present study populations.^67,72,73^ These symptoms may manifest as developmental delays, cognitive deficits, and other neurodevelopmental disorders, which are of particular concern in the case of individuals exposed to mercury during critical periods of brain development.

Given the essential role of biotinidase in biotin recycling and metabolic regulation, these findings highlight the potential health risks associated with chronic exposure to mercury in the riverside communities of the Amazon region and reinforce the need for further investigation into biotinidase as a biomarker of the effects of mercury toxicity. Longitudinal studies that monitor both enzymatic activity and clinical health outcomes over time would help to establish whether the inhibition of biotinidase has any direct, significant impact on the health of the residents of communities exposed to mercury contamination.

Overall, then, the potential consequences of the inhibition of biotinidase activity underscore the importance of monitoring exposure to mercury, especially in vulnerable riverside communities in the Amazon River Basin. Effective public health policies that reduce the contamination of the environment with mercury will be critical to ensure that risks to public health are minimized over the long term. More effective environmental monitoring and stricter regulations on mercury emissions will play a pivotal role in protecting these communities from further harm.

## 4 Conclusion

This study provides converging evidence from field, in vitro, and in silico findings that biotinidase is a direct molecular target of methylmercury. Riverine populations chronically exposed to mercury showed significantly reduced biotinidase activity, and controlled assays confirmed a clear dose-dependent inhibition of the enzyme. Molecular docking analysis further showed a specific interaction of MeHg with Cys245 at the catalytic site, providing a basis for the observed loss of activity. Taken together, these findings establish biotinidase inhibition as a biomarker reflecting the biological effects of mercury exposure and support its application in monitoring environmentally vulnerable populations.

## 5 Material and Methods

### 5.1 Materials

Sodium phosphate monobasic, sodium phosphate dibasic, ethylenediaminetetracetic acid (EDTA), trichloroacetic acid (TCA), ammonium sulfamate, sodium nitrite, *N*-(1-naphthyl) ethylenediamine dihydrochloride, bovine albumin serum (BSA), and *N*-(+)-biotinyl-4-aminobenzoic acid (B-PABA) were purchased from Sigma-Aldrich (St. Louis, MO, USA).

### 5.2 Study Populations

Volunteers from four riverside communities from the eastern Amazon region were included in the present work: São Luís do Tapajós (SLT) and Barreiras (BRR) are located in the Tapajós River basin and Panacauera (PNC) and Tucuruí Lake (TUC) are located in the Tocantins River basin (Figure 6). All four of them are typical Amazonian riverside communities with very similar lifestyles, with subsistence being based on fishing and small-scale farming. The residents of three of these populations (SLT, BRR, and TUC) are known to have been exposed to mercury through the consumption of contaminated fish.^6–8,10^

**Figure 6:**
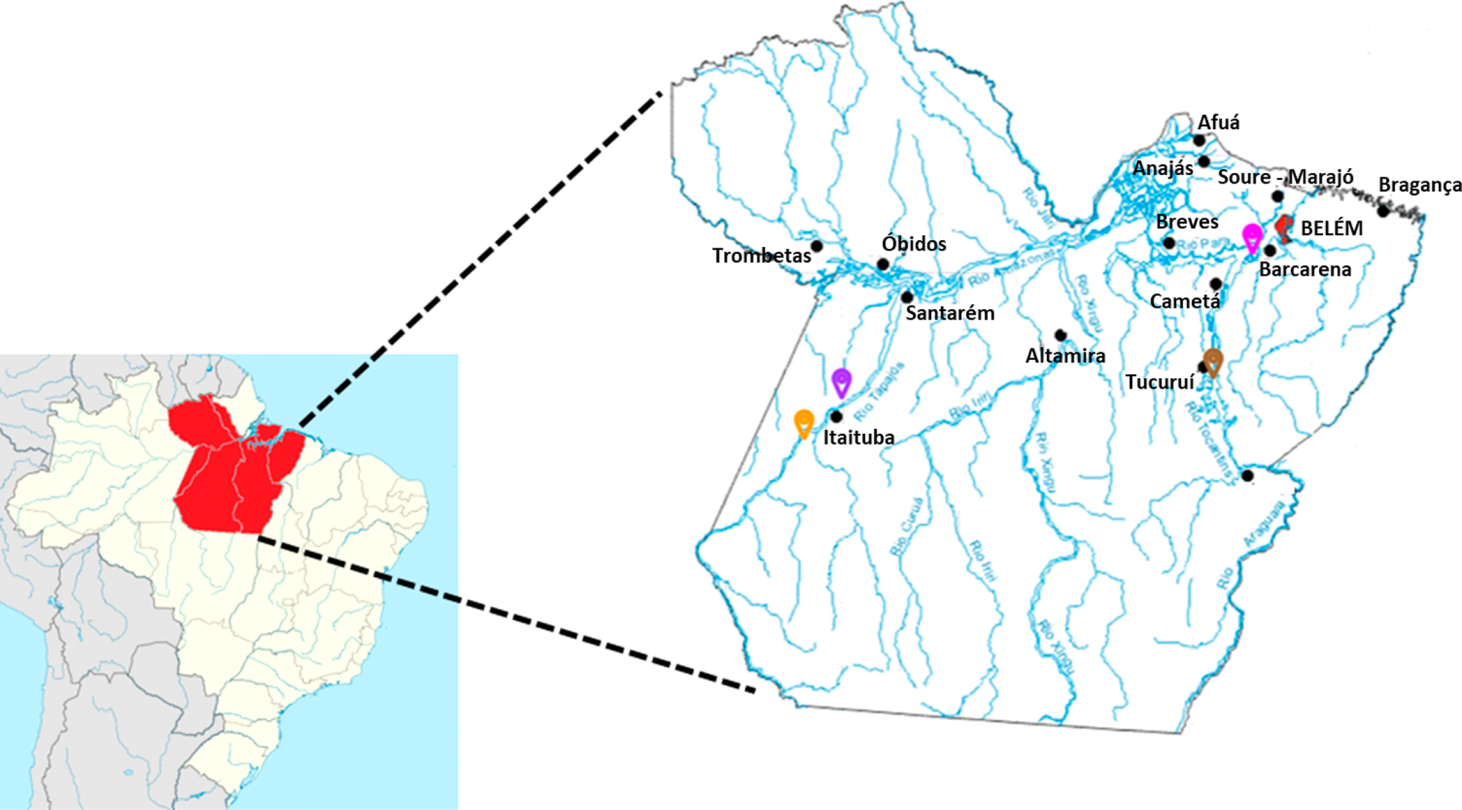
Brazilian state of Pará, showing tributaries of the Amazon River basin (blue lines), the capital (red marker), some cities (black points), and the study areas on the Tapajós River and Tocantins River (location markers). The specific locations are the communities of Panacauera (pink), São Luís do Tapajós (orange), Barreiras (purple), and Tucuruí Lake (brown). The map was adapted from public-domain sources available at Wikimedia Commons (https://commons.wikimedia.org/) and Brasil Turismo (https://www.brasil-turismo.com/mapas/para.htm).

### 5.3 Ethical Aspects

This study was conducted in accordance with the ethical principles for human research outlined in Resolution 164/86 of the National Health Council of the Brazilian Ministry of Health and was approved by the Comissão Nacional de Ética em Pesquisa (CONEP/Brazil, CAAE no. 43927115.4.0000.0018). All the participants also provided their informed consent, and their decisions were respected to ensure that no physical harm or mental trauma occurred during the collection of the blood and hair samples.

### 5.4 Blood Sampling and Hair Sampling

Blood samples were collected from healthy adult volunteers and centrifuged at 3000 rpm for 10 min. Each sample was extracted from the freshly drawn whole blood and stored at −20 °C until analysis.

Approximately 1 g of hair samples was collected from the occipital region of the head (1–2 cm from the scalp) using clean stainless-steel scissors and was stored in paper envelopes at room temperature until analysis.

### 5.5 Serum Biochemistry for Liver Function

Alanine aminotransferase (ALT), aspartate aminotransferase (AST), and gamma-glutamyl transferase (GGT) levels were analyzed in serum using standard kits (Doles©, Brazil) via spectrophotometry (Bioplus 2000).

### 5.6 Assay of Biotinidase Activity

Biotinidase activity was determined colorimetrically by quantifying the liberation of *p*-aminobenzoic acid (PABA) from N-biotinyl-*p*-aminobenzoic acid (B-PABA), using a modified version of the method described by Wolf et al.^21^ A sample of 50 μL of serum was incubated with 400 μL of substrate solution (0.15 mM B-PABA in 0.05 M sodium phosphate buffer, 0.1 mM EDTA, and 0.25% BSA, pH 6.5). A blank of each sample was prepared by inactivating the serum in a water bath at 60 °C for 30 min. The samples were incubated at 38 °C for 30 min, with the reaction being terminated by adding 100 μL of 30% TCA, after which they were homogenized in a vortex.

The homogenized samples were centrifuged at 3000 rpm for 5 min at 4 °C, and aliquots of 200 μL of the supernatant were transferred to a 96-well plate. At room temperature, 25 μL of 0.1 N sodium nitrite, 25 μL of 0.5% ammonium sulfamate, and 25 μL of 0.1% N^–1^-naphthylethylenediamine dihydrochloride were added to each well consecutively at 3 min intervals. Following a final rest interval of 3 min, the absorbance of the samples was measured using a microplate reader at λ = 546 nm. Biotinidase activity was calculated by applying correction factors for the dilution, incubation time, and the molar extinction coefficient of PABA and was expressed in nmol of PABA released per mL of serum per minute (nmol·mL^–1^·min^–1^).

### 5.7 Total Mercury Quantitation in Human Hair

Total mercury concentration was determined in hair samples using a GC-pyro AFS system according to Arrifano et al.^11^ The ERM-DB001 (human hair) certified reference material from the Institute of Reference Materials and Measurement (IRMM) was used to validate the method.

### 5.8 In Vitro Assay of the Effects of Exposure to Metals on Biotinidase Activity

For reference, blood samples were collected from healthy donors not residing in riverside communities, and the serum was separated by centrifugation at 3000 rpm for 10 min. Samples of the serum were incubated together with increasing concentrations of MeHg, FeCl_2_, CuCl_2_, and ZnCl_2_ (0.005, 0.05, 0.5, 5, and 50 μM) at room temperature for 1 h. A control group (0 μM) was also included. These concentrations were based on the environmental exposure levels recorded in contaminated populations.^74^ Biotinidase activity was measured using a spectrophotometric assay with a B-PABA substrate, as described in Section 2.4, above.

### 5.9 Computational Methods

First, the NCBI database^75^ was searched for human biotinidase, which was identified as AAC04318.1 (GI: 468824). As its three-dimensional structure has not yet been described or published, homology modeling was used to construct a 3D model.^76,76–82^ PDB code 4CYF was used as the template, with a sequence identity of 44.20%. The structure of the MeHg molecule was optimized by using the extra Avogadro function.^35^ The MeHg structure was then optimized fully using the Gaussian 09 software^83^ at the M062x/LANL2DZ level,^84^ and the results (Figure 7) were forwarded for docking.

**Figure 7:**
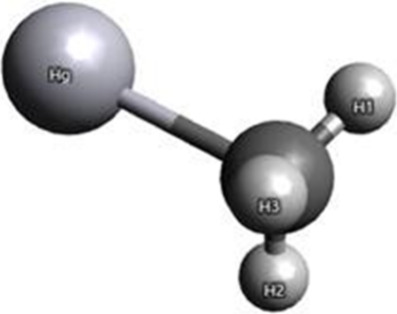
Three-dimensional structure of methylmercury, showing the optimized three-dimensional structure of MeHg^+^.

The Molegro Virtual Docker, MVD,^85^ was then used to dock MeHg into the constraint of the active biotinidase site modeled here,^86^ with Cys245, Lys212, and Glu112, using a 3Å radius at *X*: 136, *Y*: 300, and *Z*: 33.5 (Figure 8). The properties of the residues in the vicinity of the cavity were held fixed.

**Figure 8:**
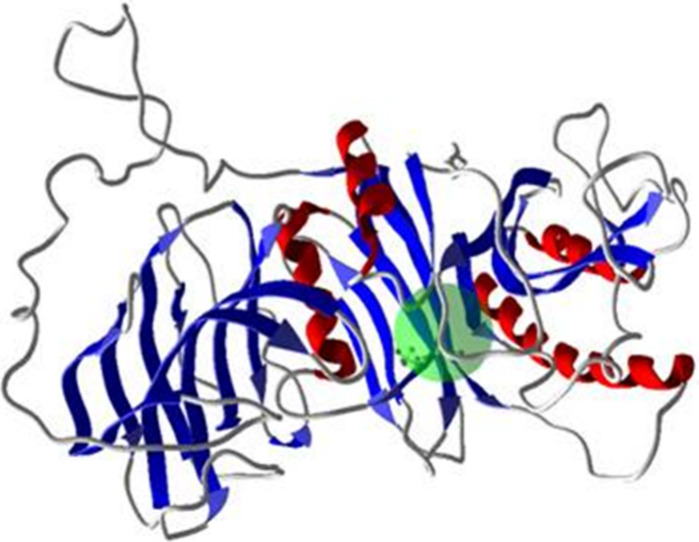
Docking constraint (green) of the active biotinidase site modeled here (α-helix in red and β-sheet in blue).

The optimized structure of the MeHg molecule was imported into the MVD as a .PDB file, and the Grid MolDock Score docking score function was applied with a 0.20 Å grid resolution, and the MolDock SE algorithm was applied in 50 runs, returning a maximum of 10 poses. These poses were saved in the mol2 format. The most representative pose was selected by its distance, the contributions of the pose energy, and the Moldock^87^ and Rerank scores.

### 5.10 Statistical Analyses

Nonparametric data were expressed as medians and interquartile ranges. The data were analyzed with a one-way ANOVA followed by Tukey’s pairwise post hoc test (parametric) or Kruskal–Wallis test followed by Dunn’s post hoc test (nonparametric) to compare groups. Differences between groups (high or low mercury) were analyzed using the Mann–Whitney test. The *P* value <0.05 was considered significant in all analyses.
